# The Potential of Multi-Task Learning in CFDST Design: Load-Bearing Capacity Design with Three MTL Models

**DOI:** 10.3390/ma17091994

**Published:** 2024-04-25

**Authors:** Zhenyu Wang, Jian Zhou, Kang Peng

**Affiliations:** School of Resources and Safety Engineering, Central South University, Changsha 410083, China; 225512075@csu.edu.cn

**Keywords:** axial compression capacity, circular concrete-filled double-skin steel tube column (CFDST), CFDST design, load-bearing capacity, multi-task learning, multi-task Lasso, variable selection and task grouping (VSTG), multi-output LS-SVR (MLS-SVR)

## Abstract

Concrete-filled double steel tubes (CFDSTs) are a load-bearing structure of composite materials. By combining concrete and steel pipes in a nested structure, the performance of the column will be greatly improved. The performance of CFDSTs is closely related to their design. However, existing codes for CFDST design often focus on how to verify the reliability of a design, but specific design parameters cannot be directly provided. As a machine learning technique that can simultaneously learn multiple related tasks, multi-task learning (MTL) has great potential in the structural design of CFDSTs. Based on 227 uniaxial compression cases of CFDSTs collected from the literature, this paper utilized three multi-task models (multi-task Lasso, VSTG, and MLS-SVR) separately to provide multiple parameters for CFDST design. To evaluate the accuracy of models, four statistical indicators were adopted (R^2^, RMSE, RRMSE, and *ρ*). The experimental results indicated that there was a non-linear relationship among the parameters of CFDSTs. Nevertheless, MLS-SVR was still able to provide an accurate set of design parameters. The coefficient matrices of two linear models, multi-task Lasso and VSTG, revealed the potential connection among CFDST parameters. The latent-task matrix *V* in VSTG divided the prediction tasks of inner tube diameter, thickness, strength, and concrete strength into three groups. In addition, the limitations of this study and future work are also summarized. This paper provides new ideas for the design of CFDSTs and the study of related codes.

## 1. Introduction

Incorporating metal components into concrete is a common approach to reinforce concrete columns [1], and concrete-filled double steel tubes (CFDSTs) have gained considerable traction in recent years. CFDSTs are a load-bearing structure produced by filling concrete between two steel tubes placed concentrically. The unique structure enables CFDST to achieve superior load-bearing capacity while being lighter in weight. Thus, CFDST is widely used in large-scale structures such as super high-rise buildings and highway bridges [2].

Load-bearing capacity [3,4], torsional behavior [2,5], bending resistance [6,7], blast resistance [8,9,10], and fire resistance [11,12] were assessed in previous studies, wherein CFDSTs with different designs (material combination or structural design) were subjected to various property trials. The experimental results showed that the design of CFDSTs has a significant impact on its properties.

As the most fundamental property of CFDSTs, the load-bearing capacity always needs to be considered. In terms of CFDST design, emphasizing on load-bearing capacity, although some international design codes already exist, such as Eurocode-4 (EC4) [13], ACI [14], and AISC [15], their reliability still remains questionable [16]. Lama et al. [17] used a non-linear finite element technique to analyze the axial compression capacity of CFDSTs made from a novel material combination. Comparing the above codes with the numerical results, it was found that EC4 and AISC were not suitable for this type of CFDSTs. Hassanein et al. [18] mentioned that the existing design codes of CFDSTs do not take the confinement effect of tubes into account. This is the reason why most models are too conservative in predicting the load-bearing capacity of CFDSTs. To avoid various constraints that make theoretical approaches more complex, machine learning techniques have been adopted, and they have achieved an accuracy far higher than EC4, ACI, and AISC [16]. In other studies, e.g., those of Tran and Kim [19], Wang et al. [4], and Chandramouli et al. [20], the prediction accuracy of the above models (EC4, ACI, and AISC) was also found to be inferior to the recently proposed methods.

However, the core idea of current studies on CFDST design is still to find a mapping from the design parameters to a certain property (e.g., load-bearing capacity) and then to find whether the design is reliable and can be verified via the prediction of this property. In a nutshell, the existing methods are more concerned about “how to verify whether a design is reliable” rather than “how to provide a reliable design directly”. Certainly, this is understandable, as the second issue is a more difficult problem involving multiple outputs.

**Figure 1:**
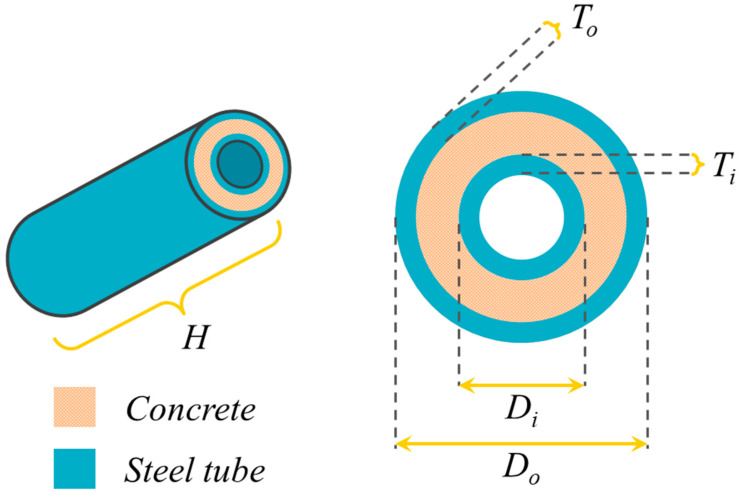
Circular cross-section of a CFDST’s structure.

Figure 1 shows the structure of a conventional CFDST. The main structural parameters include the diameters (*D*) and thicknesses (*t*) of the steel tubes, as well as column length (*H*). Among them, the length of the column is often constant in a specific project. Therefore, the design is usually focused on dimensions and properties of steel tubes, as well as the mechanical properties of concrete. Providing diverse guidance for CFDST design is simultaneously a challenge for the aforementioned methods.

As a branch of machine learning technique, multi-task learning (MTL) has been widely used in fields such as stellar spectra parameterization [21], disease cognitive scores [22], and short-term wind speed prediction [23]. By summarizing the potential correlations of multiple tasks, MTL has shown good performance in solving various multi-output problems. Therefore, using the MTL technique to guide the structural design of CFDSTs is a promising topic. In addition, using the non-linear finite element technique to verify the reliability of CFDST design is often complex. For slender structures and thin-walled structures, the accuracy requirements for modeling and solving vary at different scales [24,25]. The calculation strategy often determines whether the model can quickly converge and accurately predict with limited computational costs on an ordinary computer [26], especially for composite structures [27] like CFDSTs. With the guidance from MTL, the unnecessary trials and errors can be avoided.

From the perspective of load-bearing capacity, this research attempted to utilize three distinct MTL models for the design of CFDSTs. Based on 227 CFDST cases with circular cross-sections collected from previous literature, the MTL models were trained to provide a reliable set of design parameters. These parameters can serve as references to achieve the desired load-bearing performance. Furthermore, several statistical indicators were used to evaluate the reliability of the provided parameters.

The main content of this paper includes the following sections: Section 2 mainly introduces the models used and explains the model development process; Section 3 presents the hyper-parameter settings and experimental results; Section 4 discusses the findings from the results and lists the limitations of the experiment, as well as discussing future work; Section 5 summarizes the content and significance of this study.

## 2. Materials and Methods

### 2.1. Multi-Task Learning Models

In the supervised learning technique, a conventional model can only learn one task during the training phase. For complex problems, they are usually decomposed into several simple problems and then solved separately using single task learning (STL) models. Multi-task learning (MTL) is another kind of supervised learning technique that can achieve inductive transfer among multiple tasks. The information-sharing mechanism enables MTL to update the parameters of all tasks in a single data traversal. More importantly, this mechanism can enhance the generalization ability of each task [28].

The design guidance for CFDST requires a model to provide multiple parameters simultaneously. These output parameters are highly correlated, and each of them can correspond to a task in MTL. This characteristic leads to the compatibility between CFDST design and the MTL technique. This section summarizes the three MTL methods used in this study.

#### 2.1.1. Multi-Task Lasso

Lasso regression is a classic linear regression model proposed by Tibshirani [29]. By introducing the *L*_1_ norm into the loss function of least squares regression, Lasso regression achieved a better generalization ability than conventional linear regression. The loss function of basic Lasso is given as follows:

(1) $$\underset{W}{\min}\;\frac{1}{2n}{\left\|Y-XW\right\|}_{2}^{2}+\lambda {\left\|W\right\|}_{1}$$

where *Y* is the *n*-dimensional output vector; *X* is the matrix of input features; *W* is the coefficient vector of the linear model; ${\left\|\cdot \right\|}_{1}$ is the *L*_1_ norm of the vector; ${\left\|\cdot \right\|}_{2}$ is the *L*_2_ norm of the vector; and *λ* is the adjustment coefficient.

As the *L*_1_ regularization term ${\left\|W\right\|}_{1}$ is introduced, sparsity constraint is imposed on the coefficient vector; thereby, the coefficients of unimportant input features will be set to 0. This characteristic also allows Lasso to be widely used in feature selection.

Multi-task Lasso is an extended version of Lasso. It was presented by Obozinski et al. [30] in the problem of multi-task feature selection. Multi-task Lasso assumes that multiple similar tasks have correlated feature selection. Introducing the *L*_2,1_ norm to replace the *L*_1_ norm in Lasso, this operation leads to information sharing among different tasks during model iteration. The loss function of multi-task Lasso is given as follows:

(2) $$\underset{W}{\min}\;\frac{1}{2n}{\left\|Y-XW\right\|}_{Fro}^{2}+\lambda {\left\|W\right\|}_{2,1}$$

where vectors *Y, X,* and *W* become matrix form; ${\left\|\cdot \right\|}_{Fro}$ is the Frobenius norm of matrix; and ${\left\|\cdot \right\|}_{2,1}$ is the *L*_2,1_ norm of matrix. The mechanism of the *L*_2,1_ norm in MTL is detailed in the work of Liu et al. [31].

#### 2.1.2. VSTG

Variable selection and task grouping (VSTG) is another MTL model developed based on linear regression [32]. In VSTG, there are two fundamental theories that need to be understood: low-rank hypothesis and structure approach. As prior knowledge, the low rank hypothesis assumes that the information required for multiple similar tasks is always redundant. For example, if two tasks are more related, their selection of input features may be more similar. Therefore, when all tasks iterate simultaneously, the matrix composed of hyper-parameters must be low rank. In MTL, low-rank constraints can encourage models to develop towards low-rank structures. The structure approach means the association between multiple tasks can be represented by a certain structure. Group structure is the most commonly used type, and it is also adopted by VSTG. In VSTG, similar tasks are considered to belong to the same group. Tasks within the same group share more information during the model development phase.

**Figure 2:**
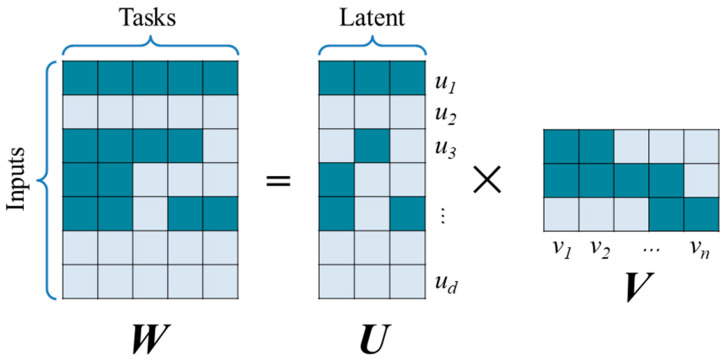
Illustration of matrix decomposition in VSTG.

VSTG decomposes the coefficient matrix, *W*, into two matrices, *U* and *V*. Moreover, latent bases are introduced for learning and describing the overlapping structures among different tasks. The matrix decomposition is shown in Figure 2, where the dark cells represent non-zero values, and light cells represent zero values.

The matrix *U* is called the variable-latent matrix, and it records the principal components of input features after the dimensionality reduction operation. The matrix *V* is called the latent-task matrix, and it records the group structure of tasks. For example, the column vectors *v*_1_ and *v*_2_ are similar in Figure 2, indicating that Task 1 and Task 2 are highly correlated. Thus, these two tasks should belong to the same group. In the training phase, two matrices *U* and *V* are updated via ADMM (alternating direction method of multipliers). The loss function of VSTG is given as follows:

(3) $$\begin{array}{c} \underset{U,V}{\min}\;\sum_{i=1}^{T}\frac{1}{2N_{i}}{\left\|\vec{y_{i}}-X_{i}U\vec{v_{i}}\right\|}_{2}^{2}\\ s.t\;{\left\|U\right\|}_{1}\leq \varphi_{1},\;{\left\|U\right\|}_{1,\;\infty }\leq \varphi_{2},\;\\ \sum_{i=1}^{T}{\left({\left\|\vec{v_{i}}\right\|}_{k}^{sp}\right)}^{2}\leq \theta \end{array}$$

where $\vec{v_{i}}$ is the *i*-th column vector in matrix *V*; *φ*_1_, *φ*_2_, and *θ* are the parameters of low-rank constraints; ${\left\|\cdot \right\|}_{1,\infty }$ is the *L*_1,∞_ norm; and ${\left\|\cdot \right\|}_{k}^{sp}$ represents the *k*-support norm.

The constraints in Equation (3) can also exist in the form of regularization terms. Thus, the problem can be transformed into a regularized objective function, as follows:

(4) $$\sum_{i=1}^{T}\frac{1}{2N_{i}}{\left\|\vec{y_{i}}-X_{i}U\vec{v_{i}}\right\|}_{2}^{2}+\lambda_{1}{\left\|U\right\|}_{1}+\lambda_{2}{\left\|U\right\|}_{1,\infty }+\mu \sum_{i=1}^{T}{\left({\left\|\vec{v_{i}}\right\|}_{k}^{sp}\right)}^{2}$$

where *λ*_1_, *λ*_2_, and *μ* are regularization parameters.

#### 2.1.3. MLS-SVR

SVR (support vector regression) is a concise and high-performance regression model [33,34]. With the maximum margin mechanism, SVR aims to find the best fitting position for linear models in the feature space. The minority vectors that determine the position of margins are called support vectors. Although SVR uses linear models for regression, it can also be applied to some complex nonlinear problems by adopting soft margin and kernel methods [35,36,37,38,39].

**Figure 3:**
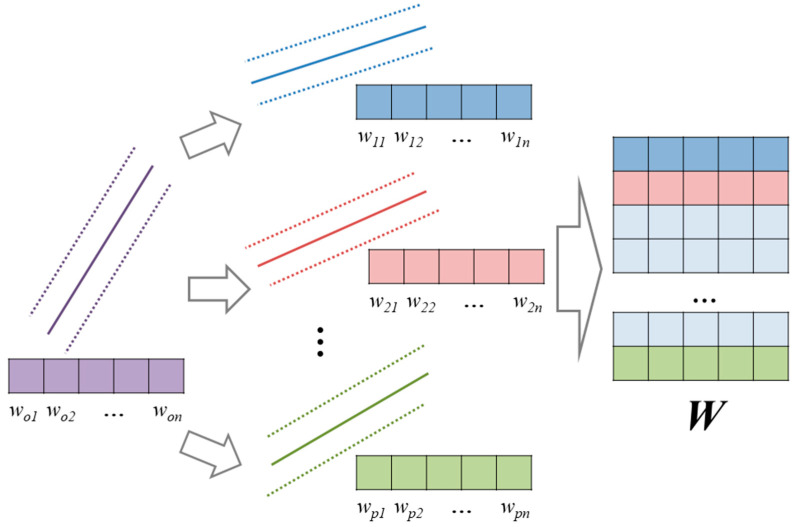
Evolution process of coefficient vectors in MLS-SVR.

MLS-SVR (multi-output least-squares SVR) is an MTL method developed based on SVR [40]. Unlike the idea of matrix decomposition in VSTG, MLS-SVR believes that the coefficient vectors of different tasks can evolve from an initial coefficient vector. Rewriting a coefficient vector $\vec{w_{p}}$ into $\vec{w_{o}}+\vec{u_{p}}$, the MTL version SVR is dedicated to learning the initial vector $\vec{w_{o}}$ and evolved component $\vec{u_{p}}$ from the dataset. Figure 3 shows the illustration of MLS-SVR’s intuition.

In MLS-SVR, the traditional SVR optimization problem has been rewritten into a matrix version, as follows:

(5) $$\begin{array}{c} \min_{{\vec{w}}_{o}\in R^{p},\Lambda \in R^{p},\vec{b}\in R^{m}}\;\frac{1}{2}{\vec{w}}_{o}^{T}{\vec{w}}_{o}+\frac{1}{2}\frac{\lambda }{m}trace(\Lambda^{T}\Lambda )+\beta \frac{1}{2}trace(\Theta^{T}\Theta )\\ s.t.\;Y=\Omega^{T}W+repmat({\vec{b}}^{T},N,1)+\Theta \end{array}$$

where $\vec{w_{o}}$ represents the initial coefficient vector; *W* is the coefficient matrix, wherein elements are $\vec{w_{p}}$; Λ is a matrix composed of $\vec{u_{p}}$; Θ denotes a matrix composed of slack variables from each task; Ω is a matrix used to map input features to higher dimensional space; $\vec{b}$ is the bias vector; *p* and *m* are the dimensions of inputs and outputs, respectively; *N* is the number of samples in training set; and *λ* and *β* are regularization parameters.

### 2.2. Database

The database used in this study comprises 227 instances of circular cross-section CFDSTs subjected to uniaxial compression [41,42,43,44,45,46,47,48,49,50,51,52,53,54,55,56,57,58,59,60]. These tests were conducted using uniaxial compression testing machines, with sensors installed on the specimens. The data generated from the experiments were automatically collected by the computer.

**Figure 4:**
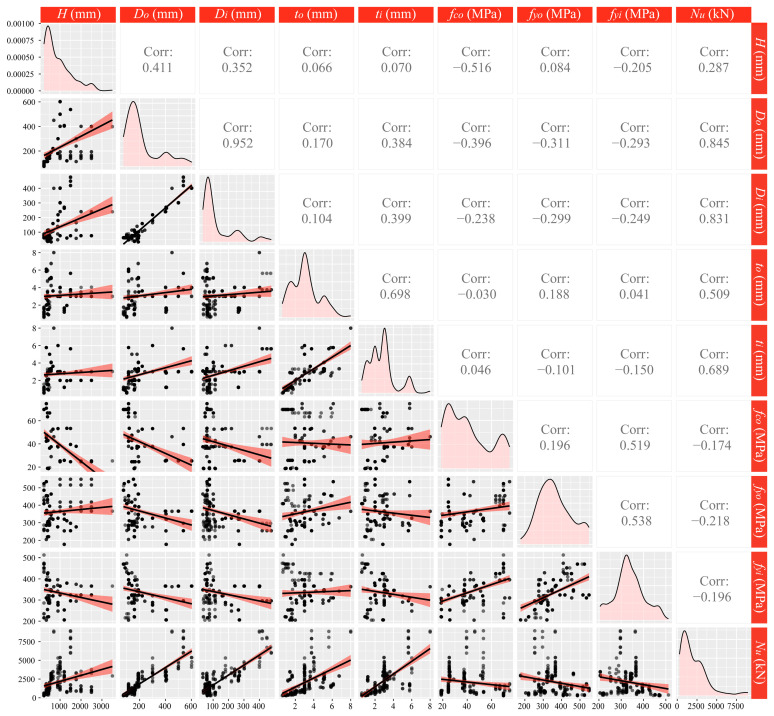
Scatter plot matrix and correlation coefficients of the database (Dots represent the samples, while lines display the distribution trend of dots).

Table 1 summarizes the 9 parameters required for CFDST design in the database. These parameters are related to the strength of materials (concrete and steel tubes), tube dimensions, and CFDST load-bearing capacity. Additionally, statistical descriptions of the database are given in Table 2. The column length ranging from 230 to 3502 mm included CFDST cases from the laboratory scale to the site scale. In Figure 4, the scatter plot matrix of the database is displayed, and the correlation coefficients between variables are calculated. There, the three parameters, diameter of the outer steel tube, diameter of the inner steel tube, and axial compression capacity of the column were highly correlated. It is worth noting that both *D_o_* and *D_i_* were positively correlated with *N_u_*. However, this makes sense, because a larger column usually means a higher strength.

### 2.3. Model Development

Because of various considerations such as materials and economy, the design of CFDSTs is often customized. In practice, a certain type of steel tube or concrete may be prioritized for use, or tubes with a specific dimension may have to be adopted due to special needs (e.g., outer diameter or self-weight of the column). Therefore, the experiments in this study only demonstrated the scenario of providing guidance on inner steel tube and concrete based on the selected outer steel tube.

The inputs and outputs are shown in Table 3. This section demonstrates the development process of the MTL models based on the example scenario. The similar development pattern can be transferred to other situations that have not been demonstrated.

Different magnitudes of variables may lead to inaccuracy for machine learning models. Especially for MTL, multiple tasks cannot be effectively trained if there is a significant difference in the units. Thus, all variables were normalized into [−10, 10]. As the values in the generally used normalization [−1, 1] are too small, [−10, 10] can enable the data-sensitive linear model to learn suitable coefficients successfully.

**Figure 5:**
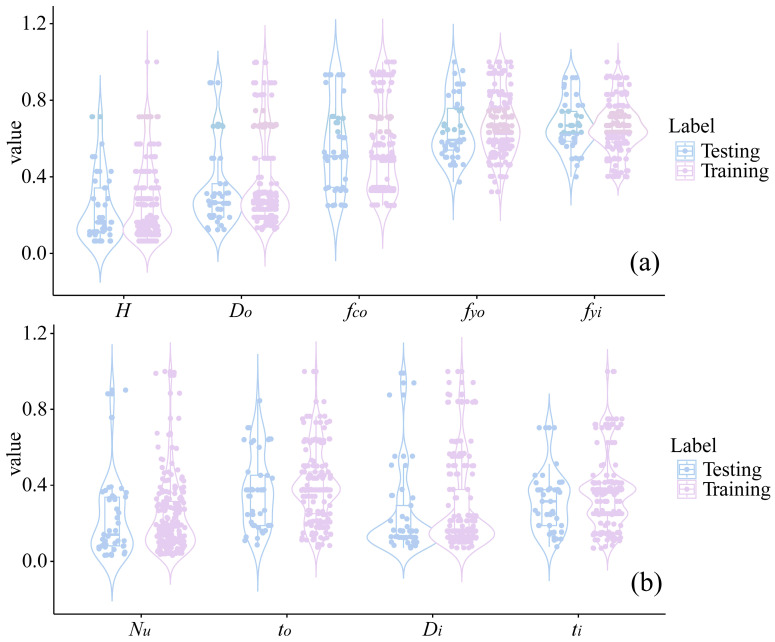
Dataset division and sample distributions in the training and testing sets. (**a**) division of parameters *H*, *D_o_*, *f_co_*, *f_yo_* and *f_yi_*; (**b**) division of parameters *N_u_*, *t_o_*, *D_i_* and *t_i_*.

All samples were allocated to two datasets, namely, the training set (80%) and the testing set (20%) [61]. The training set was used for model development, while the model validation was conducted via a testing set. In supervised learning techniques, there is an assumption that samples and populations follow the same distribution. Therefore, the model development phase requires that the distributions of the training and testing sets are as similar as possible. This partitioning strategy that aims to obtain similar training and testing sets ensures the reliability of model evaluation provided in the testing phase. The distributions of two datasets are shown in Figure 5.

**Figure 6:**
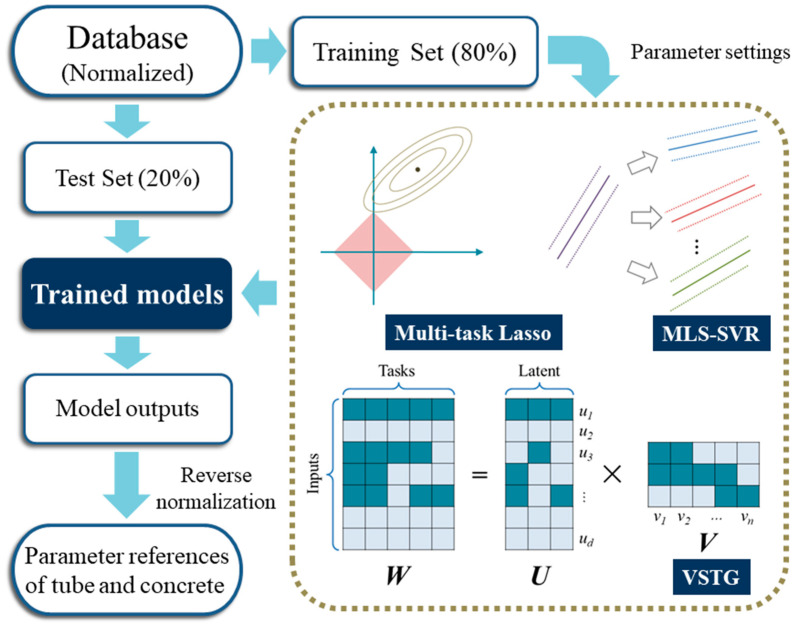
Flowchart of model development.

Figure 6 shows the entire model development process. Based on the training set, three kinds of MTL models were iterated separately. Subsequently, the testing set was input into these models, and several statistical indicators were adopted to analyze the accuracy of the results.

To evaluate the reliability of the provided parameters, 4 statistical indicators were introduced. Table 4 provides the definitions and expressions of these indicators. The *R^2^* is generally used to measure the degree of correlation between measurements and predictions [62,63]; RMSE and RRMSE are two indicators used to characterize the error between measurements and predictions [64,65,66,67,68,69]; and *ρ* is an indicator that comprehensively considers correlation and error [70,71]. The larger the *ρ* value, the less accurate the model.

## 3. Results

By adjusting and testing, hyper-parameters of three MTL models were obtained separately. With the hyper-parameter settings given in Table 5, three MTL models completed iterations. For each MTL model, the same development process was repeated five times to mitigate errors caused by randomness. All the experimental results are provided in Appendix A.

However, the performance of the two linear models on the tasks *f_yi_* and *f_co_* was not satisfactory enough. Table 6 shows a set of results that was relatively good in experiments of multi-task Lasso (experiment 1), while Table 7 shows an acceptable set of results in VSTG experiments (experiment 1). Compared to strength tasks (*f_yi_* and *f_co_*), multi-task Lasso and VSTG seemed better at the predictions of dimension tasks (*D_i_* and *t_i_*). As shown in Table 6 and Table 7, the R^2^ values of strength tasks were at a low level whether on the training set or the testing set. For task *f_yi_*, the R^2^ was always less than 0.4, and that means the provided *f_yi_* was weakly correlated with the actual value. For task *f_co_*, two linear models seemed ineffective even on the training set.

Unlike the previous two models, MLS-SVR showed quite good performance in all tasks, and the average result of five experiments is displayed in Table 8. From the perspective of task *D_i_*, the RMSE and RRMSE values on the testing set were reduced by 40% compared to multi-task Lasso and VSTG. The RMSE and RRMSE of task *t_i_* also decreased by 20%. Moreover, all the *ρ* values were below 0.1.

**Figure 7:**
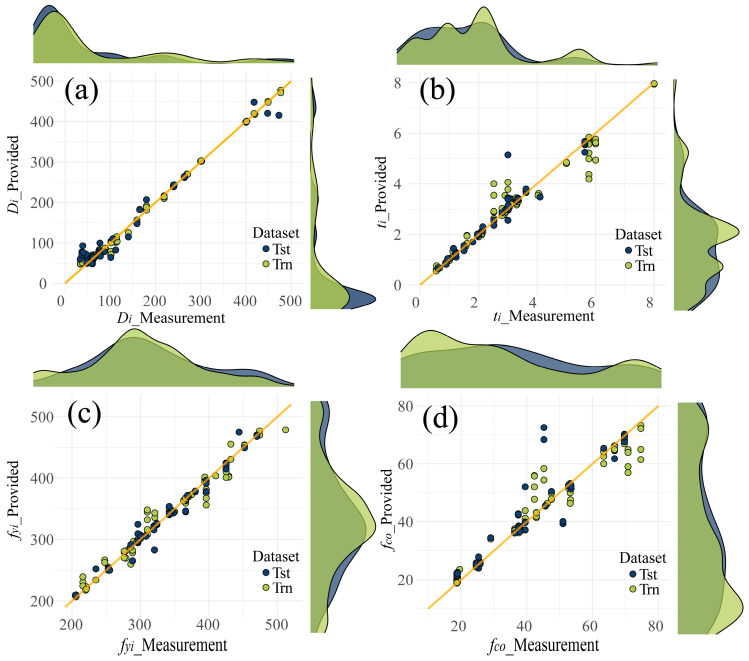
Scatter plots of the provided parameters by MLS-SVR: (**a**) *D_i_* plot; (**b**) *t_i_* plot; (**c**) *f_yi_* plot; (**d**) *f_co_* plot.

**Figure 8:**
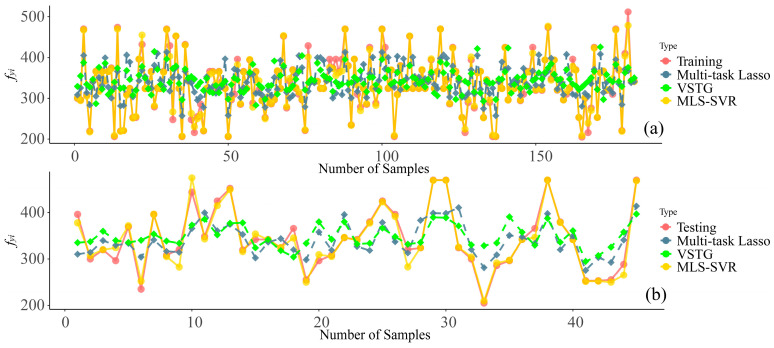
Illustrations of the comparison among MTL provided and the actual values on task *f_yi_*: (**a**) training set; (**b**) testing set.

**Figure 9:**
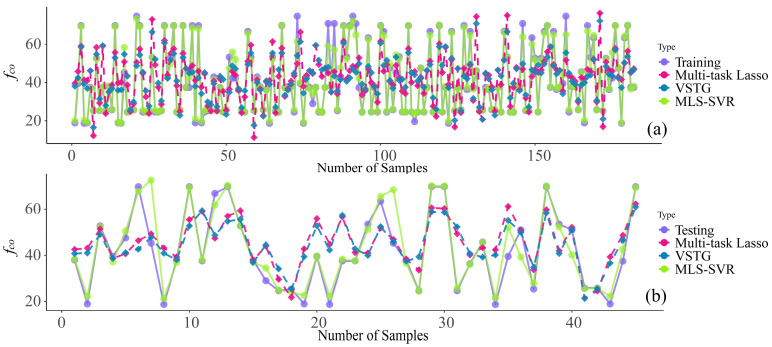
Illustrations of the comparison among MTL provided and actual values on task *f_co_*: (**a**) training set; (**b**) testing set.

Figure 7 displays the scatter plots of predictions provided by MLS-SVR (from experiment 4). Even if there were some outliers in the task *t_i_* (within [2, 4] and [5, 6]) and *f_co_* (within [40, 50]) tasks, the distribution of scattering points still showed the accuracy of MLS-SVR in providing parameters for CFDST design. In Figure 8 and Figure 9, the performances of all MTL models in task *f_yi_* and *f_co_* are compared. It can be noticed that the predictions of the two linear models were conservative in these two unsatisfactory tasks. In other words, the predicted values of two linear models showed the same trend as the actual values increased or decreased, but the degree was not significant.

## 4. Discussion

This section mainly clarifies the problems presented in Section 3, and their causes are also elucidated. Moreover, the feature matrices learned by two linear models are analyzed. Additionally, the limitations and future work are listed at the end of this section.

### 4.1. Nonlinearity

In the tasks of *f_yi_* and *f_co_*, linear models were unable to effectively learn the task. Overfitting occurred in both multi-task Lasso and VSTG. Based on this fact, there were two possible causes:

- Cause A: The provided input features did not contain the key information related to the strength tasks. For task *f_yi_* and *f_co_*, all input features were useless.
- Cause B: There was a certain non-linear relationship between input features and strength tasks. Thereby, linear models were unable to simulate this nonlinearity well.

Cause A can be ruled out, or rather, it cannot be the main issue. This is because the MLS-SVR (with kernel methods for nonlinearity) was able to achieve very accurate predictions in these two tasks, using the same input features.

**Figure 10:**
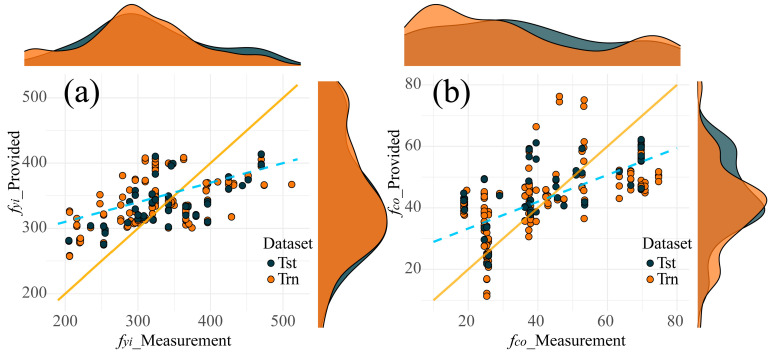
Scatter plots of strength parameters provided by multi-task Lasso: (**a**) *f_yi_* plot; (**b**) *f_co_* plot.

**Figure 11:**
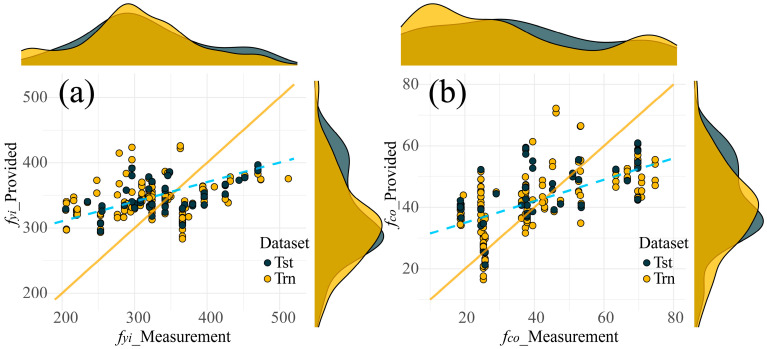
Scatter plots of strength parameters provided by VSTG: (**a**) *f_yi_* plot; (**b**) *f_co_* plot.

**Figure 12:**
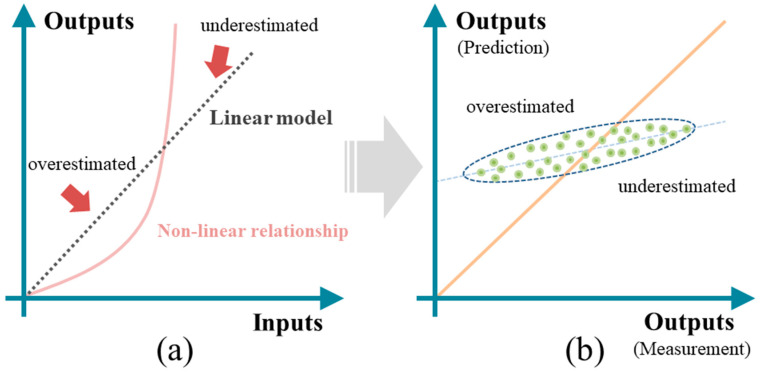
The impact of nonlinearity on linear models: (**a**) a concave nonlinear function; (**b**) misguided scatter distribution.

To further verify cause B, the scatter plots of strength tasks from multi-task Lasso and VSTG are shown in Figure 10 and Figure 11, respectively. In both task *f_yi_* and *f_co_*, the direction of scatter distribution (blue lines) tended to be horizontal, and that was evidence of nonlinearity between inputs and tasks. For example, if a linear model was used to fit a concave nonlinear function (as shown in Figure 12a), the distribution of scatter would appear in the pattern shown in Figure 12b. With a constant as the boundary, the predicted values within smaller-scale regions were often overestimated, while the predictions within larger-scale regions were often underestimated. Additionally, the more horizontal scatter distribution also indicated the reason why the predictions of two linear models were more conservative in Figure 8 and Figure 9.

### 4.2. Model Interpretability

Although the two linear models did not excel in all tasks, they had better interpretability. The coefficient matrices (*W*) obtained from two linear models, as well as variable-latent matrix (*U*) and latent-task matrix (*V*) provided by VSTG, all as interpretable components contained the information that revealed the potential correlations among CFDST parameters.

**Figure 13:**
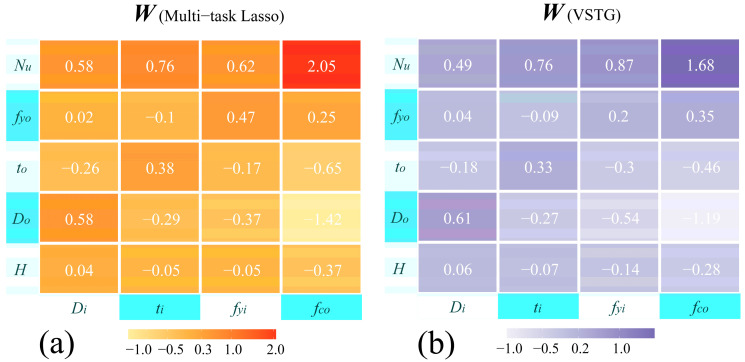
The coefficient matrices obtained from two linear models: (**a**) *W* of multi-task Lasso; (**b**) *W* of VSTG.

Two coefficient matrices from multi-task Lasso and VSTG are shown in Figure 13. Firstly, the load-bearing capacity (axial compression capacity, *N_u_*) was highly correlated with each output feature, with the strength of concrete contributing the most. Secondly, the contributions of *H* and *f_yo_* to task *D_i_* and task *t_i_* were close to 0. That means the influence of CFDST length and outer tube strength on the inner tube dimension was minimal.

For tasks *f_yi_* and *f_co_*, there was almost no obvious sparsity in the coefficient vectors (except for the coefficient of *H* in task *f_yi_* in multi-task Lasso). This phenomenon indicated that all inputs were indispensable for both tasks. However, these two models were still unable to fit tasks *f_yi_* and *f_co_* well. Therefore, it is reasonable to infer that there must have been a lack of certain features, or that potential information had not been fully explored. These inferences were mentioned and answered in Section 4.1. Nevertheless, it is noteworthy that the coefficient vector of task *f_yi_* indeed had a relatively stronger sparsity (smaller coefficients) than task *f_co_*’s. Perhaps this was the reason why the scatter distribution of task *f_yi_* in Figure 10 and Figure 11 was more concentrated, as the dimension of input feature space of task *f_yi_* was compressed more effectively.

**Figure 14:**
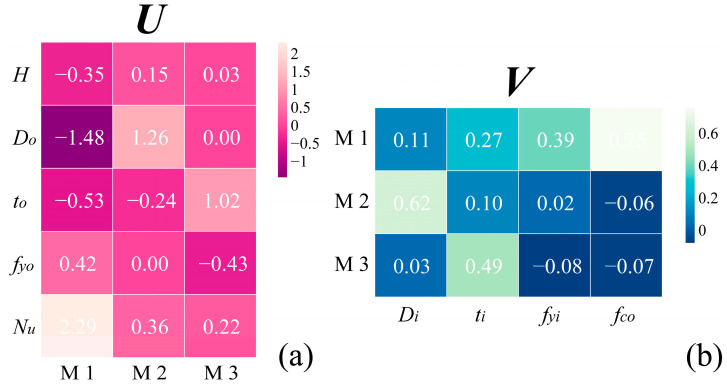
Two matrices provided by VSTG: (**a**) variable-latent matrix (*U*); (**b**) latent-task matrix (*V*).

The variable-latent matrix *U* and latent-task matrix *V* from VSTG are shown in Figure 14. The matrix *U* recorded the mapping from the original input space to the lower dimensional feature space. Figure 14a shows that the input features *D_o_* and *f_yo_* were able to be expressed using only two bases in the new feature space, and the projection of feature *H* on the third base M3 was also almost 0 (Figure 14a). Excess information from the original five-dimensional input space was still able to be fully expressed after being compressed into three-dimensional space. The matrix *V* is a coefficient matrix used to map the new feature space to the output space. This matrix also stores the group structure among multiple tasks. Seemingly, these tasks can be divided into three groups:

- Group 1 (task *D_i_*): The coefficient of the second base M2 is significantly higher than the other two;
- Group 2 (task *t_i_*): The coefficient of the second base M2 is significantly lower than the other two;
- Group 3 (task *f_yi_* and *f_co_*): The first base is obviously important, while the values of the other two are almost 0.

This indicates that the diameter and thickness of the inner steel tube are two completely different tasks (their feature selections are opposite), while the strength of the inner steel tube and concrete are very similar tasks.

### 4.3. Limitations and Future Work

Although the correlation of models with experimental results demonstrated the potential applications of MTL techniques in guiding CFDST design, there are still many limitations:

- The samples collected in the database were all CFDSTs with circular cross-sections, as this shape is the most conventional. CFDSTs of other shapes may have more parameters, so their design must be more complex.
- MTL is a data-driven approach, and the performance of this technique depends on the quantity and quality of data. Currently, the uniaxial compression cases of CFDSTs are sufficient, while cases of other property trials are still lacking.
- The interpretability of linear models can reveal the potential connection among CFDST parameters. However, this connection is purely mathematical, and the mechanism behind it still remains a mystery.

This study was conducted in a specific condition, and further validations of “whether MTL techniques would be applicable in other situations of CFDST design” will be necessary. As an auxiliary tool, the linear MTL models own good interpretability, and that allows MTL to play a role in the future development of CFDST design codes. In addition, the mechanistic research on CFDSTs (load bearing, failure, etc.) will continue to be crucial. Experiments and cases on the properties of CFDSTs are still lacking.

## 5. Conclusions

Due to the fact that previous methods can only verify the reliability of a CFDST design and cannot provide direct parameter guidance, this paper proposed using the MTL technique to guide CFDST design. The main works and findings are as follows:

- With 227 uniaxial compression cases of CFDSTs collected from previous literature, three kinds of MTL models were trained to provide multiple parameters for CFDST design. Based on a specific application scenario, the development process of the MTL models was demonstrated.
- During the testing phase, MLS-SVR was able to accurately provide reliable CFDST parameters, while the other two linear models, multi-task Lasso and VSTG, were unable to provide valuable parameters of inner steel tube strength and concrete strength.
- The distribution of scattered points reflected the potential nonlinearity in the task *f_yi_* and *f_co_*, and the connotation in scatter distribution was discussed in detail. Furthermore, the coefficient matrices of two linear models and the potential group structure among the CFDST parameters were clarified.
- At the end of Section 4, the limitations of the study and future work are also summarized.

In conclusion, the MTL technique has great potential in guiding CFDST design. With a set of directly provided parameters, the workload of engineers in CFDST design will be greatly reduced. Due to the interpretability, linear MTL models can also serve as an analytical tool and assist in the study of the property mechanisms and design standards of CFDSTs.

## Figures and Tables

**Table 1:** Parameter definitions and descriptions.

Variables	Descriptions	Note
*H* (mm)	Column length	External dimensions
*D_o_* (mm)	Diameter of the outer steel tube	
*D_i_* (mm)	Diameter of the inner steel tube	Internal dimension
*t_o_* (mm)	Thickness of the outer steel tube	Steel tube thickness
*t_i_* (mm)	Thickness of the inner steel tube	
*f_co_* (MPa)	Peak strength of unconfined cylinder (*d* × *h* = 150 mm × 300 mm)	Concrete strength
*f_yo_* (MPa)	Yield strength of the outer steel tube	Steel tube strengths
*f_yi_* (MPa)	Yield strength of the inner steel tube	
*N_u_* (kN)	Axial compression capacity	Load-bearing capacity

**Table 2:** Statistical description of parameters in the database.

Parameters	Mean	Median	St. D *	Min	Max
*H* (mm)	877.830	572.000	149.429	230.000	3502.000
*D_o_* (mm)	220.778	165.100	32.152	74.700	603.400
*D_i_* (mm)	129.618	76.000	25.742	33.500	477.000
*t_o_* (mm)	3.097	3.000	0.350	0.590	8.000
*t_i_* (mm)	2.735	2.850	0.335	0.550	8.000
*f_co_* (MPa)	40.813	37.500	4.094	18.700	74.700
*f_yo_* (MPa)	362.696	350.000	20.736	177.000	549.000
*f_yi_* (MPa)	335.807	324.000	15.434	205.000	512.000
*N_u_* (kN)	2095.051	1574.000	413.341	283.000	8950.000

* Note: St. D—standard deviation.

**Table 3:** The inputs and outputs in the example scenario.

Inputs	Outputs
*H*, *D_o_*, *t_o_*, *f_yo_*, *N_u_*	*D_i_*, *t_i_*, *f_yi_*, *f_co_*

**Table 4:** Statistical indicators for evaluating the reliability of the provided parameters.

Statistical Indicators	Expressions
Coefficient of determination (R^2^)	$\frac{{\left[\sum_{i=1}^{N}\left(M_{i}-\bar{M_{i}}\right)\left(P_{i}-\bar{P_{i}}\right)\right]}^{2}}{\sum_{i=1}^{N}{\left(M_{i}-\bar{M_{i}}\right)}^{2}\sum_{i=1}^{N}{\left(P_{i}-\bar{P_{i}}\right)}^{2}}$
Root mean square error (RMSE)	$\sqrt{\frac{\sum_{i=1}^{N}{\left(M_{i}-P_{i}\right)}^{2}}{N}}$
Relative root mean squared error (RRMSE)	$\frac{RMSE}{\bar{P_{i}}}$
Performance index (*ρ*)	$\frac{RRMSE}{1+\sqrt{R^{2}}}$

Note: M—measurement value; P—reference value provided by the model.

**Table 5:** The hyper-parameter settings used for the experiments.

MTL Models	Hyper-Parameters	Values
Multi-task Lasso	Regularization parameter *λ*	0.091
VSTG	Regularization parameter *λ*_1_	0.15
	Regularization parameter *λ*_2_	0.14
	Regularization parameter *μ*	1.25
	*k* (*k*-support norm)	2
	Number of latent bases *M*	3
MLS-SVR	Regularization parameter *λ*	2.013
	Regularization parameter *β*	33.571
	Kernel function	ERBF
	Kernel function parameter *σ*	4.493

**Table 6:** Statistical evaluation of multi-task Lasso.

Outputs	Dataset	R^2^	RMSE	RRMSE	*ρ*
*D_i_*	Trn	0.926	30.542	0.240	0.122
	Tst	0.904	34.905	0.275	0.141
*t_i_*	Trn	0.648	0.866	0.312	0.173
	Tst	0.896	0.497	0.179	0.092
*f_yi_*	Trn	0.258	56.611	0.166	0.11
	Tst	0.457	49.811	0.146	0.087
*f_co_*	Trn	0.382	13.915	0.306	0.189
	Tst	0.389	13.627	0.299	0.184

Note: Trn—training set; Tst—testing set.

**Table 7:** Statistical evaluation of VSTG.

Outputs	Dataset	R^2^	RMSE	RRMSE	*ρ*
*D_i_*	Trn	0.922	31.210	0.245	0.125
	Tst	0.900	35.709	0.280	0.144
*t_i_*	Trn	0.646	0.870	0.308	0.171
	Tst	0.898	0.513	0.182	0.093
*f_yi_*	Trn	0.136	61.320	0.176	0.128
	Tst	0.296	56.909	0.163	0.106
*f_co_*	Trn	0.355	14.190	0.317	0.199
	Tst	0.390	13.473	0.301	0.186

Note: Trn—training set; Tst—testing set.

**Table 8:** Statistical evaluation of MLS-SVR.

Outputs	Dataset	R^2^	RMSE	RRMSE	*ρ*
*D_i_*	Trn	0.998	5.265	0.044	0.022
	Tst	0.966	20.287	0.169	0.085
*t_i_*	Trn	0.973	0.239	0.093	0.047
	Tst	0.906	0.392	0.152	0.078
*f_yi_*	Trn	0.987	7.518	0.022	0.011
	Tst	0.965	12.633	0.037	0.019
*f_co_*	Trn	0.975	2.754	0.064	0.032
	Tst	0.866	6.499	0.150	0.078

Note: Trn—training set; Tst—testing set.

## Data Availability

The data that support the findings of this study are available on request from the corresponding author upon reasonable request.

## Appendix A

**Table A1:** All experimental results from multi-task Lasso.

Outputs	Dataset	R^2^	RMSE	RRMSE	*ρ*
*D_i_*	Trn_experiment_1	0.925798241	30.54154609	0.240264793	0.12244763
	Trn_experiment_2	0.925798326	30.54154601	0.240265398	0.122447936
	Trn_experiment_3	0.925798412	30.54154593	0.240266004	0.122448242
	Trn_experiment_4	0.925798497	30.54154587	0.24026661	0.122448548
	Trn_experiment_5	0.921823761	31.23514035	0.24540618	0.125199791
	Trn_mean	0.925003447	30.68026485	0.241293797	0.122998429
	Tst_experiment_1	0.903852591	34.90496	0.274591	0.140764
	Tst_experiment_2	0.903852949	34.90481	0.27459	0.140764
	Tst_experiment_3	0.903853308	34.90466	0.27459	0.140764
	Tst_experiment_4	0.903853667	34.90451	0.27459	0.140764
	Tst_experiment_5	0.898420822	35.83429	0.28154	0.144539
	Tst_mean	0.902766668	35.09065	0.27598	0.141519
*t_i_*	Trn_experiment_1	0.647650602	0.865708461	0.312352516	0.173070786
	Trn_experiment_2	0.647650541	0.86570846	0.312353227	0.173071183
	Trn_experiment_3	0.647650481	0.865708459	0.312353938	0.173071581
	Trn_experiment_4	0.64765042	0.865708458	0.312354649	0.173071978
	Trn_experiment_5	0.642129795	0.87523493	0.30854615	0.171287964
	Trn_mean	0.646546368	0.867613754	0.311592096	0.172714698
	Tst_experiment_1	0.895809286	0.497392	0.179462	0.092198
	Tst_experiment_2	0.895808763	0.497392	0.179462	0.092199
	Tst_experiment_3	0.89580824	0.497392	0.179463	0.092199
	Tst_experiment_4	0.895807718	0.497392	0.179463	0.092199
	Tst_experiment_5	0.896319131	0.522132	0.184067	0.094551
	Tst_mean	0.895910627	0.50234	0.180383	0.092669
*f_yi_*	Trn_experiment_1	0.257824781	56.61053299	0.166176103	0.110213567
	Trn_experiment_2	0.257826623	56.61053286	0.166176058	0.110213404
	Trn_experiment_3	0.257828465	56.61053274	0.166176012	0.110213242
	Trn_experiment_4	0.257830307	56.61053264	0.166175967	0.110213079
	Trn_experiment_5	0.143605727	61.05929462	0.175032617	0.12693149
	Trn_mean	0.234983181	57.50028517	0.167947351	0.113556956
	Tst_experiment_1	0.457067241	49.81142	0.146218	0.087239
	Tst_experiment_2	0.457071203	49.81112	0.146217	0.087238
	Tst_experiment_3	0.457075164	49.81082	0.146216	0.087237
	Tst_experiment_4	0.457079126	49.81052	0.146215	0.087237
	Tst_experiment_5	0.309128976	56.65101	0.162396	0.104368
	Tst_mean	0.427484342	51.17898	0.149452	0.090664
*f_co_*	Trn_experiment_1	0.382329222	13.91468957	0.305536581	0.188797713
	Trn_experiment_2	0.382332284	13.91468953	0.305535146	0.188796538
	Trn_experiment_3	0.382335346	13.91468949	0.305533712	0.188795363
	Trn_experiment_4	0.382338409	13.91468947	0.305532277	0.188794187
	Trn_experiment_5	0.349522654	14.25304033	0.319577939	0.20084028
	Trn_mean	0.375771583	13.98235968	0.308343131	0.191204816
	Tst_experiment_1	0.388893448	13.62725	0.299225	0.184296
	Tst_experiment_2	0.388897421	13.62727	0.299224	0.184295
	Tst_experiment_3	0.388901394	13.62728	0.299223	0.184294
	Tst_experiment_4	0.388905367	13.62729	0.299222	0.184293
	Tst_experiment_5	0.383469564	13.54646	0.303735	0.187578
	Tst_mean	0.387813439	13.61111	0.300126	0.184951

**Table A2:** All experimental results from VSTG.

Outputs	Dataset	R^2^	RMSE	RRMSE	*ρ*
*D_i_*	Trn_experiment_1	0.922420	31.20567	0.244677	0.124808
	Trn_experiment_2	0.921584	31.29855	0.245879	0.125449
	Trn_experiment_3	0.921693	31.27291	0.245672	0.12534
	Trn_experiment_4	0.921771	31.25197	0.245519	0.125259
	Trn_experiment_5	0.921824	31.23514	0.245406	0.125200
	Trn_mean	0.921858	31.25285	0.245431	0.125211
	Tst_experiment_1	0.899683	35.70926	0.279989	0.143693
	Tst_experiment_2	0.898060	35.89606	0.281997	0.144788
	Tst_experiment_3	0.898216	35.87198	0.281801	0.144681
	Tst_experiment_4	0.898334	35.85119	0.281651	0.144599
	Tst_experiment_5	0.898421	35.83429	0.28154	0.144539
	Tst_mean	0.898543	35.83256	0.281396	0.14446
*t_i_*	Trn_experiment_1	0.64555	0.870096	0.308432	0.171022
	Trn_experiment_2	0.642602	0.874941	0.308653	0.171319
	Trn_experiment_3	0.642569	0.874921	0.308611	0.171298
	Trn_experiment_4	0.642411	0.875007	0.308572	0.171286
	Trn_experiment_5	0.642130	0.875235	0.308546	0.171288
	Trn_mean	0.643052	0.874040	0.308563	0.171243
	Tst_experiment_1	0.897740	0.513348	0.181972	0.093439
	Tst_experiment_2	0.897543	0.518708	0.182985	0.093964
	Tst_experiment_3	0.897429	0.51924	0.183152	0.094053
	Tst_experiment_4	0.896939	0.520482	0.183548	0.094269
	Tst_experiment_5	0.896319	0.522132	0.184067	0.094551
	Tst_mean	0.897194	0.518782	0.183145	0.094055
*f_yi_*	Trn_experiment_1	0.136017	61.32048	0.175747	0.128395
	Trn_experiment_2	0.142423	61.08823	0.175156	0.127165
	Trn_experiment_3	0.142487	61.08705	0.175144	0.127149
	Trn_experiment_4	0.142957	61.07670	0.175097	0.127057
	Trn_experiment_5	0.143606	61.05929	0.175033	0.126931
	Trn_mean	0.141498	61.12635	0.175235	0.127339
	Tst_experiment_1	0.295738	56.90902	0.163104	0.10565
	Tst_experiment_2	0.305408	56.78643	0.162821	0.104868
	Tst_experiment_3	0.306126	56.75469	0.162723	0.104760
	Tst_experiment_4	0.307393	56.71041	0.162579	0.104591
	Tst_experiment_5	0.309129	56.65101	0.162396	0.104368
	Tst_mean	0.304759	56.76231	0.162725	0.104847
*f_co_*	Trn_experiment_1	0.355196	14.18623	0.317429	0.198892
	Trn_experiment_2	0.349209	14.26040	0.319686	0.200941
	Trn_experiment_3	0.349363	14.25735	0.319629	0.200889
	Trn_experiment_4	0.349489	14.25495	0.319587	0.200850
	Trn_experiment_5	0.349523	14.25304	0.319578	0.200840
	Trn_mean	0.350556	14.24239	0.319182	0.200483
	Tst_experiment_1	0.389745	13.47301	0.301470	0.185600
	Tst_experiment_2	0.383357	13.55413	0.303853	0.187661
	Tst_experiment_3	0.383610	13.54961	0.303762	0.187581
	Tst_experiment_4	0.383550	13.54818	0.303742	0.187574
	Tst_experiment_5	0.383470	13.54646	0.303735	0.187578
	Tst_mean	0.384746	13.53428	0.303312	0.187199

**Table A3:** All experimental results from MLS-SVR.

Outputs	Dataset	R^2^	RMSE	RRMSE	*ρ*
*D_i_*	Trn_experiment_1	0.997956	4.950633	0.04127	0.020645
	Trn_experiment_2	0.998098	4.774466	0.039817	0.019918
	Trn_experiment_3	0.99828	4.541585	0.037884	0.01895
	Trn_experiment_4	0.99679	6.21027	0.051617	0.025829
	Trn_experiment_5	0.997152	5.847266	0.04864	0.024337
	Trn_mean	0.997655	5.264844	0.043846	0.021936
	Tst_experiment_1	0.966752	20.16726	0.168119	0.08477
	Tst_experiment_2	0.967065	20.06718	0.167353	0.084377
	Tst_experiment_3	0.96684	20.11983	0.16783	0.084623
	Tst_experiment_4	0.965648	20.61851	0.171372	0.086435
	Tst_experiment_5	0.966064	20.46098	0.170203	0.085836
	Tst_mean	0.966474	20.28675	0.168976	0.085208
*t_i_*	Trn_experiment_1	0.977555	0.221349	0.086169	0.043329
	Trn_experiment_2	0.977566	0.221087	0.086098	0.043293
	Trn_experiment_3	0.980432	0.206541	0.080415	0.040406
	Trn_experiment_4	0.963974	0.280518	0.109174	0.055088
	Trn_experiment_5	0.967474	0.266504	0.103741	0.0523
	Trn_mean	0.9734	0.2392	0.093119	0.046883
	Tst_experiment_1	0.901291	0.402229	0.156584	0.080326
	Tst_experiment_2	0.901474	0.401589	0.156391	0.080223
	Tst_experiment_3	0.898315	0.408791	0.159159	0.081712
	Tst_experiment_4	0.916494	0.368955	0.143593	0.073361
	Tst_experiment_5	0.912926	0.376779	0.146668	0.075004
	Tst_mean	0.9061	0.391669	0.152479	0.078125
*f_yi_*	Trn_experiment_1	0.989376	6.803098	0.019868	0.009961
	Trn_experiment_2	0.989653	6.711025	0.019602	0.009826
	Trn_experiment_3	0.99114	6.20726	0.018126	0.009083
	Trn_experiment_4	0.980407	9.245705	0.027043	0.013588
	Trn_experiment_5	0.982968	8.620965	0.025204	0.012656
	Trn_mean	0.986709	7.517611	0.021969	0.011023
	Tst_experiment_1	0.965078	12.61071	0.036829	0.018578
	Tst_experiment_2	0.965056	12.62965	0.036889	0.018608
	Tst_experiment_3	0.965206	12.59698	0.036785	0.018555
	Tst_experiment_4	0.964875	12.67916	0.037085	0.018708
	Tst_experiment_5	0.964985	12.64765	0.036977	0.018653
	Tst_mean	0.96504	12.63283	0.036913	0.018621
*f_co_*	Trn_experiment_1	0.977615	2.637616	0.060853	0.030599
	Trn_experiment_2	0.978519	2.583422	0.059613	0.029968
	Trn_experiment_3	0.980582	2.456158	0.056692	0.028485
	Trn_experiment_4	0.969402	3.094903	0.071324	0.035939
	Trn_experiment_5	0.97116	3.000334	0.069161	0.034833
	Trn_mean	0.975456	2.754487	0.063529	0.031965
	Tst_experiment_1	0.865928	6.502839	0.15003	0.077713
	Tst_experiment_2	0.865614	6.515199	0.15034	0.077881
	Tst_experiment_3	0.86555	6.522601	0.150551	0.077992
	Tst_experiment_4	0.865382	6.473133	0.149178	0.077284
	Tst_experiment_5	0.865663	6.478995	0.149347	0.077365
	Tst_mean	0.865628	6.498553	0.149889	0.077647
